# The G Allele and GG Genotype of the Junctional Cadherin 5 Associated (JCAD) Is a Biomarker Predicting Myocardial Infarction in Slovenian Subjects with Type 2 Diabetes Mellitus

**DOI:** 10.3390/genes16111378

**Published:** 2025-11-11

**Authors:** Miha Tibaut, Danijel Petrovič

**Affiliations:** 1 Department of Internal Medicine, General Hospital Rakičan, Ulica dr. Vrbnjaka 6, 9000 Murska Sobota, Slovenia; 2 Institute of Histology and Embryology, Faculty of Medicine, University of Ljubljana, Korytkova 2, 1105 Ljubljana, Slovenia; danijel.petrovic@mf.uni-lj.si; 3 International Centre for Cardiovascular Diseases MC Medicor d.d., 6310 Izola, Slovenia

**Keywords:** myocardial infarction, type 2 diabetes mellitus, JCAD, KIAA1462, polymorphism, coronary artery disease

## Abstract

Background: Patients with type 2 diabetes mellitus (T2DM) have a two- to fourfold higher risk of myocardial infarction (MI), yet genetic determinants of this excess risk remain incompletely defined. The *JCAD* (junctional cadherin 5 associated; formerly *KIAA1462*) locus has been implicated in coronary artery disease through genome-wide association studies, but data in diabetic populations are scarce. Objectives: To assess whether the rs3739998 polymorphism of *JCAD* is associated with MI in Slovenian subjects with T2DM and to explore its relationship with coronary disease burden and coronary artery calcium (CAC). Methods: We performed a retrospective cross-sectional association study of 1471 Slovenian subjects with T2DM: 387 with prior MI and 1084 without clinical evidence of coronary artery disease. Genotyping for *JCAD* rs3739998 was performed using a fluorescence-based competitive allele-specific PCR (KASPar). A coronary computed tomographic angiography (CCTA) substudy (*n* = 146) evaluated the number of diseased coronary arteries, stenosis severity, and CAC score. Results: The GG genotype was more frequently observed in MI cases compared to controls in unadjusted analysis (OR 1.37; *p* = 0.05) but association was lost with adjustment for confounders (GG vs. CC, aOR 1.63, *p* = 0.09). The G allele was also more prevalent among cases (OR 1.18; *p* = 0.05, unadjusted analysis). In the CCTA substudy, no significant associations were observed between rs3739998 and the number of diseased vessels, stenosis grade, or CAC. Conclusions: In a Slovenian T2DM cohort, the *JCAD* rs3739998 G allele and GG genotype showed a nominal association with prior MI that did not persist after multivariable adjustment. There was no clear relationship with anatomic disease burden or CAC, underscoring the need for replication in larger cohorts and functional studies to clarify the mechanism and clinical utility.

## 1. Introduction

### 1.1. Type 2 Diabetes Mellitus and Cardiovascular Risk

Type 2 diabetes mellitus (T2DM) is one of the most prevalent metabolic disorders worldwide, affecting an estimated 9% of the global adult population. According to the World Health Organization, diabetes accounted directly for 1.5 million deaths in 2012 [1], and cardiovascular disease represents the major driver of morbidity and mortality among patients with T2DM. In Slovenia, approximately 167,300 individuals live with diabetes, with annual healthcare costs attributable to diabetes representing 5.2% of total expenditures and rising at a rate of 3–5% annually [2].

The risk of coronary artery disease (CAD) is two- to fourfold higher in patients with T2DM than in the nondiabetic population [3]. This increased susceptibility is not fully explained by conventional risk factors such as dyslipidemia, hypertension, and smoking. Instead, chronic hyperglycemia, insulin resistance, systemic inflammation, and oxidative stress create a pro-atherogenic environment that accelerates vascular damage [4].

### 1.2. Pathophysiology of Atherosclerosis in T2DM

The atherosclerotic process is driven by retention and modification of lipoproteins, endothelial activation, recruitment of inflammatory cells, and smooth muscle cell proliferation. In T2DM, hyperglycemia promotes the formation of advanced glycation end-products, which further stimulate oxidative stress and inflammation. Oxidative stress, resulting from overproduction of reactive oxygen species and reactive nitrogen species, plays a central role in endothelial dysfunction, low-density lipoprotein (LDL) oxidation, and plaque progression [4].

While traditional risk factors and metabolic abnormalities accelerate plaque formation, genetic predisposition significantly influences the interindividual variability in susceptibility to myocardial infarction (MI) among patients with T2DM [5]. Genome-wide association studies (GWAS) have identified over 58 loci associated with CAD, many of which are related to vascular remodeling, endothelial function, and inflammatory signaling [6].

### 1.3. The Role of JCAD in Vascular Biology

The Junctional Cadherin 5 Associated (JCAD) gene, also known as *KIAA1462*, is located on chromosome 10p11.23 and encodes a protein expressed in endothelial cells, where it localizes at intercellular junctions. JCAD interacts with vascular endothelial-cadherin and other junctional proteins, contributing to the structural integrity and signaling capacity of the endothelium [7]. Functional studies suggest that JCAD is involved in angiogenesis, endothelial cell migration, and maintenance of barrier function [8].

GWAS have consistently linked *JCAD* polymorphisms to CAD risk across diverse populations [9,10]. Although the exact mechanisms by which *JCAD* variants contribute to cardiovascular risk are not fully understood, disruption of endothelial junctional signaling is believed to increase vascular permeability, leukocyte infiltration, and plaque instability. Additionally, JCAD has been involved in Hippo signaling pathways and in regulating endothelial apoptosis and proliferation, which are processes closely related to vascular remodeling [8].

### 1.4. The rs3739998 Polymorphism of JCAD

Among the polymorphisms within the *JCAD* locus, rs3739998 has gained attention for its potential role in CAD susceptibility. Located in the gene’s third exonic region, rs3739998 represents a missense variant [11], thereby affecting JCAD function. While previous studies have shown associations between *JCAD* variants and CAD in general populations, their significance in diabetic cohorts has not been thoroughly explored.

In T2DM, where endothelial dysfunction and accelerated atherogenesis are key features of disease progression, genetic variants like rs3739998 may have a significant impact on MI risk. Recognizing such polymorphisms is both pathophysiologically and clinically important, as they could serve as biomarkers for early risk assessment or even as therapeutic targets.

## 2. Rationale for the Study

Although there is strong epidemiological evidence linking T2DM with higher MI risk, the role of genetic polymorphisms is not fully understood in certain populations. Slovenia, being a genetically fairly homogeneous Central European group, is ideal for genetic association studies.

The present study was embedded within a broader doctoral research project investigating the role of oxidative stress-related genes in MI susceptibility among Slovenian patients with T2DM. In addition to genes such as *ROMO1*, *NQO1*, *GCH1*, *RAC1*, *CDKN2B-AS1*, and *PHACTR1*, we specifically evaluated the rs3739998 polymorphism of *JCAD*.

## 3. Materials and Methods

In this retrospective cross-sectional association study, we included patients with T2DM with a disease duration of more than ten years. All participants were of Caucasian descent and unrelated. Ethnicity was considered important, as the distribution of genotypes for various polymorphisms frequently differs across populations.

All patients provided written informed consent to participate in the study and for blood sampling for molecular genetic testing. Additionally, they completed a standardized questionnaire that covered personal and family medical history, as well as clinical data.

Patients were recruited during routine visits at outpatient clinics for internal medicine, cardiology, and diabetology at the General Hospitals Murska Sobota, Izola, and Slovenj Gradec, as well as at MC Medicor, the University Medical Centre Maribor, and the University Medical Centre Ljubljana.

All data were stored in a secure database at the Institute of Histology and Embryology, Faculty of Medicine, University of Ljubljana. The study was approved by the National Medical Ethics Committee of the Ministry of Health of the Republic of Slovenia at its meeting on 18 July 2017 (approval number 0120-372/2017).

### 3.1. Subjects

We included 1471 subjects with T2DM. All participants were divided into two groups: a group of 387 patients with a history of MI (MI group) and a group of 1084 subjects without clinical evidence of coronary artery disease, based on medical history, electrocardiography, transthoracic echocardiography, and submaximal exercise testing (control group). Control status was defined clinically, and coronary imaging was not required, nor were coronary artery calcium (CAC) scores collected controls. All subjects were of Central European ancestry and recruited from tertiary and secondary healthcare centers across Slovenia.

Subjects were classified as having T2DM based on the current criteria of the American Diabetes Association [12]. The diagnosis of MI was made according to the universal definition of myocardial infarction [13]. Patients with MI were enrolled in the study nine months after the acute event.

Clinical and demographic data were gathered at enrollment, including age, sex, body mass index (BMI), smoking status, arterial hypertension, dyslipidemia, and duration of diabetes. A detailed medical history, including previous MI, was recorded.

Importantly, this was a within-T2DM case–control study and did not include an external MI group without diabetes; therefore, inferences are restricted to MI susceptibility among individuals with T2DM.

In addition to the primary cohort, a coronary computed tomographic angiography (CCTA) substudy was performed. A total of 146 patients with T2DM underwent CCTA based on clinical indications. The assessment included the extent of coronary artery involvement, the number of coronary arteries with significant stenosis, and the coronary calcium burden, quantified by the CAC score. The inclusion criteria for this substudy were identical to those used in the primary cohort, except that these patients reported exertional chest pain during outpatient visits, while transthoracic echocardiography and submaximal exercise testing yielded normal results.

### 3.2. Biochemical Analyses

Serum levels of glucose, creatinine, total cholesterol, high-density lipoprotein (HDL) cholesterol, and triglycerides were determined in all participants using standard colorimetric assays on an automated biochemical analyzer (Ektachem 250 Analyzer, Eastman Kodak Company, Rochester, MN, USA). Serum LDL cholesterol levels were calculated using the Friedewald formula. Hyperlipidemia was defined as total cholesterol > 5 mmol/L and/or triglycerides > 2 mmol/L, or as treatment with lipid-lowering medication.

Glycated hemoglobin (HbA1c) (nondiabetic reference range 3.8–5.3%) was measured by high-performance liquid chromatography, with the mean of the three most recent HbA1c values recorded for each individual. High-sensitivity C-reactive protein (hsCRP) was measured using a latex-enhanced immunonephelometric assay.

### 3.3. Genotyping

Genomic DNA was isolated from 100 μL of whole peripheral blood using a Qiagen isolation kit(Qiagen Ltd., Manchester, UK). The polymorphism rs3739998 (JCAD) was genotyped at the Institute of Histology and Embryology, Faculty of Medicine, University of Ljubljana, using a fluorescence-based competitive allele-specific PCR system (KASPar). Details of the method are available at: http://www.kbioscience.co.uk/.

### 3.4. CT Imaging

Patients included in the second part of the study underwent CCTA and CAC scoring at the International Centre for Cardiovascular Diseases MC Medicor, Izola, and at the General Hospital Murska Sobota. Noninvasive visualization of the epicardial coronary artery system and detection of stenoses were performed using a dual-energy CT scanner (Siemens, Munich, Germany). CT angiograms were independently interpreted by two senior radiologists. The CCTA substudy included T2DM patients referred for clinically indicated imaging with normal TTE and exercise testing; it was not designed or powered to compare MI-T2DM versus T2DM, and analyses were performed across rs3739998 genotypes within T2DM.

Normal coronary arteries were defined as the absence of obstructive atherosclerotic plaques in the epicardial coronary tree. Non-obstructive CAD was defined as the presence of plaque involving <50% of the vessel cross-sectional area. The severity of CAD was graded according to the degree of luminal narrowing (<50%, ≥50–≤75%, and >75%) and according to the number of diseased vessels (graded from 0 to 3; 0 = absence of obstructive CAD, 1 = single-vessel disease, 2 = two-vessel disease, 3 = three-vessel disease).

When the left main coronary artery (LMCA) was diseased, it was scored as 2, and subsequent involvement of both its main branches—the left anterior descending (LAD) and the left circumflex (LCx)—was not counted separately. In the absence of obstructive LMCA lesions, each affected branch (LAD and/or LCx) was assigned 1 point. Disease in the right coronary artery (RCA) was scored as 1.

For the CAC assessment, all lesions with an attenuation exceeding 130 Hounsfield units (HU) were included. The area of each lesion (mm^2^) was multiplied by a factor determined by the maximum HU value of that lesion. 130–199 HU: factor 1; 200–299 HU: factor 2; 300–399 HU: factor 3; ≥400 HU: factor 4. The total CAC score was calculated as the sum of values for all lesions.

### 3.5. Statistical Methods

Normally distributed continuous variables were expressed as mean ± standard deviation, while skewed variables were expressed as median (interquartile range, IQR). The normality of distribution was tested using the Kolmogorov–Smirnov test. For normally distributed continuous variables, the unpaired Student’s *t*-test was applied; for non-normally distributed variables, the Mann–Whitney U test was used. Discrete variables were compared using Pearson’s χ^2^ test, which was also used to test for deviations from Hardy–Weinberg equilibrium in genotype distributions. For contingency tables with expected cell counts < 5, Fisher’s exact test was used to determine the significance of associations between categorical variables. Associations between the number of diseased coronary arteries and the degree of luminal narrowing were evaluated using Pearson’s χ^2^ test. Fisher’s exact test was applied when cell counts were <5. Variables: male sex, waist circumference, diastolic blood pressure, fasting glucose, total cholesterol, HDL and LDL cholesterol, and physical activity were included in a stepwise multivariable logistic regression model. Diabetes duration was considered clinically important but methodologically sensitive and was pre-specified to be excluded from the primary adjustment. This exclusion is because it reflects time since diagnosis (a proxy that may misrepresent cumulative glycemic exposure and vary by MI-related diagnostic timing), is strongly collinear with age and HbA1c/fasting glucose, and risks over-adjustment. A sensitivity analysis was performed, adjusting additionally for diabetes duration. A *p*-value ≤ 0.05 was considered statistically significant. Statistical analyses were performed using SPSS version 26 (SPSS Inc., Chicago, IL, USA).

## 4. Results

### 4.1. Study Population

The clinical characteristics and biochemical parameters of the study cohort are presented in Table 1.

Cases (*n* = 387, patients with MI) were more often male, had smaller waist circumferences, and better-controlled diastolic blood pressure. They also had higher total cholesterol and LDL cholesterol levels, and lower HDL cholesterol levels. The duration of diabetes was shorter in MI patients, while the mean fasting plasma glucose level was higher. A higher proportion of MI patients had experienced a stroke and reported being more physically active.

The two groups were similar regarding age, body mass index, systolic blood pressure, smoking status, HbA1c, triglycerides, and hsCRP. They also had comparable rates of arterial hypertension and transient ischemic attack. In the MI group, a higher proportion of patients were treated with statins and lipid-lowering medications, while a lower proportion received oral antidiabetic drugs or insulin therapy.

The clinical characteristics and biochemical parameters of the Slovenian T2DM population from CCTA substudy are shown in Table 2. Patients were predominantly male and overweight. Most had arterial hypertension, while a smaller proportion were smokers.

### 4.2. Polymorphism rs3739998 of the JCAD Gene

The genotype and allele frequencies of the *JCAD* rs3739998 polymorphism are presented in Table 3. Genotype distributions in both MI patients and controls were consistent with Hardy–Weinberg equilibrium (HWE) (MI patients: *p* = 0.141; controls: *p* = 0.152). Genotype frequencies in controls (*p* = 0.79) did not significantly differ from those reported in the 1000 Genomes Project Phase 3 European population.

The GG genotype was significantly more frequent among MI patients (OR 1.37, *p* = 0.05). Similarly, the G allele was more prevalent in this group (OR 1.18, *p* = 0.05) (Table 3).

We performed binary logistic regression under different genetic models of rs3739998 (Table 4). No significant associations were found between genotypes or alleles and MI risk in Slovenian T2DM patients. However, a trend toward increased risk with the GG genotype, particularly in the codominant model, was observed. Odds ratios were adjusted for variables significant in univariate analyses (male sex, waist circumference, diastolic blood pressure, fasting glucose, total cholesterol, HDL and LDL cholesterol, and physical activity). While the unadjusted model suggested a borderline association (GG vs. CC), no significant association was observed after adjustment (Table 4). The higher adjusted point estimate relative to the unadjusted odds ratio is compatible with the removal of negative confounding by clinical covariates. Conversely, inclusion of multiple, intercorrelated predictors (lipid measures, anthropometrics, blood pressure, glycemia, physical activity) increased the standard error, widening the confidence interval and yielding a higher non-significant *p*-value.

In the subgroup of T2DM patients who underwent CCTA, no statistically significant differences in rs3739998 genotypes or alleles were observed in relation to the number of diseased coronary arteries (Table 5) or the degree of luminal narrowing (Table 6).

When evaluating CAC scores, a numerical trend toward higher values in carriers of the GG genotype and G allele was observed; however, these differences did not reach statistical significance (Table 7).

## 5. Discussion

### 5.1. Study Population and Risk Factors for Myocardial Infarction

Our study included 387 MI patients and 1084 controls with T2DM. The mean age was comparable between groups (~65 years), consistent with age being the strongest determinant of cardiovascular risk. As expected, a significantly higher proportion of MI patients were male, reflecting the well-established sex difference in MI incidence.

Obesity was prevalent in both groups, with BMI values at the threshold of overweight and class I obesity. While BMI did not differ significantly, central obesity is known to correlate more strongly with MI [14]. Hypertension was common, but only diastolic blood pressure differed, being lower in MI patients, likely due to stricter clinical monitoring.

MI patients had higher total and LDL cholesterol levels and lower HDL cholesterol levels, consistent with dyslipidemia as a key risk factor in atherosclerosis. They also had higher fasting glucose and shorter diabetes duration, indicating poorer metabolic control prior to the event.

Smoking prevalence was relatively low but numerically higher among MI patients, while stroke was also more common, reflecting the systemic nature of atherosclerosis. Interestingly, MI patients reported more frequent physical activity, possibly reflecting lifestyle changes or participation in rehabilitation after MI.

Overall, the clinical profile of our MI patients aligns with established cardiovascular risk factors, supporting the validity of our cohort for genetic association analyses.

### 5.2. Polymorphism rs3739998 of the JCAD Gene

To date, no study has investigated the association of *JCAD* polymorphisms with coronary pathology in the Slovenian population. In unadjusted analyses, rs3739998 showed a borderline association with prior MI within T2DM (GG vs. CC: OR 1.37, *p* = 0.05; G allele: OR 1.18, *p* = 0.05). However, this association did not remain significant after multivariable adjustment (GG vs. CC: aOR 1.63, 95% CI 0.93–2.87; *p* = 0.09). The higher adjusted point estimate relative to the crude odds ratio is consistent with the removal of negative confounding. However, the inclusion of several intercorrelated clinical predictors (lipid measures, glycemia, blood pressure, anthropometrics, physical activity) increased the standard error, widening the confidence interval and attenuating statistical significance. Accordingly, our primary inference is based on the adjusted, non-significant result. A sensitivity model additionally adjusting for diabetes duration yielded materially unchanged estimates (direction preserved; non-significant).

The *JCAD* gene (formerly *KIAA1462*) was initially linked to extreme obesity in mammals. It was first named junctional protein associated with coronary artery disease, after two large GWAS identified its association with coronary artery disease [9,10] and because of its localization at endothelial cell–cell junctions [15]. It was later renamed junctional cadherin 5 associated due to its close localization to cadherin 5 in adherens junctions. Localization in endothelial adherens junctions was confirmed by Shigeoka et al. [16] in samples of human submandibular glands. They also observed preferential expression in arteries and microcirculatory regions affected by inflammation. *JCAD* is strongly expressed in tissues rich in endothelial cells, including the aorta, lungs, brain, and coronary arteries [7].

*JCAD* encodes a ~145 kDa protein lacking clearly recognizable functional domains and showing limited homology to other protein families [17]. Its function remains incompletely understood but is under active investigation. Hara et al. [18] reported reduced tumor growth and intratumoral neovascularization in JCAD-deficient mice following injection of tumor cells, suggesting a role in pathological but not developmental angiogenesis. Subsequent research showed that JCAD inhibits Hippo signaling, leading to increased activity of the transcription factor Yap [8]. This, in turn, enhances proinflammatory signaling pathways, endothelial cell proliferation and migration, angiogenesis, and monocyte adhesion to endothelial cells [8]. Xu et al. [7] confirmed that JCAD-deficient mice exhibit reduced vascular inflammation, less atherosclerosis, and improved endothelium-dependent vasodilation. JCAD has also been implicated in mechanotransduction of shear stress, as JCAD-deficient mice developed less atherosclerosis on the inner curvature of the aortic arch [17]. Expression of *JCAD* is strongly influenced by blood flow and is highest at sites of turbulent or nonlaminar flow [7,17]. In the same models, reduced expression of proinflammatory adhesion molecules ICAM and VCAM-1 was observed. Collectively, these findings suggest that JCAD contributes to the pathophysiology of coronary artery disease by activating proinflammatory pathways and promoting atherogenesis.

Attention to *JCAD* increased after GWAS demonstrated its association with coronary artery disease and MI [9,10]. Several polymorphisms have been identified, including rs3739998, a missense variant resulting from substitution of the C allele with the G allele. *JCAD* polymorphisms have also been linked to pulmonary emphysema [19], Alzheimer’s disease [20], ovarian tumors [21], and progression of nonalcoholic steatohepatitis to hepatocellular carcinoma [22]. While the rs3739998 polymorphism has been replicated in several GWAS of coronary artery disease [23,24], only one association study confirmed this finding [25]. Conversely, some studies failed to show associations with coronary artery disease [26,27] or MI [11]. According to the initial GWAS [9], each risk allele at this locus increases the risk of coronary artery disease or MI by ~15%, amounting to ~30% in homozygotes. These findings align with our results, where the presence of the G allele increased MI risk by 18% and the GG genotype by 37%. However, our study did not show a correlation with the extent of coronary disease or CAC score. Our data indicate that JCAD may increase susceptibility to myocardial infarction without a corresponding rise in coronary calcification or angiographic disease burden, pointing to mechanisms related to plaque vulnerability or thrombogenicity rather than sheer atherosclerotic volume. This interpretation aligns with findings by Liberale et al. [28], who showed that JCAD promotes arterial thrombosis via PI3K/Akt signaling. Activation of this pathway suppresses procoagulant expression. Reduced *JCAD* expression results in diminished activation of the coagulation cascade (decreased tissue factor expression and activity) and enhanced fibrinolysis (reduced expression of plasminogen activator inhibitor-1). These findings were further supported by higher plasma levels of JCAD, tissue factor, and plasminogen activator inhibitor-1. Larger, adequately powered cohorts are needed to confirm these relationships and clarify their clinical relevance.

The likelihood of MI increases with the presence of vulnerable plaques characterized by a thin fibrous cap, macrophage infiltration and inflammation, reduced smooth muscle cell content, a large lipid-rich necrotic core, intraplaque hemorrhage, neovascularization, adventitial inflammation, and high tissue factor content [29]. Given the role of JCAD in promoting inflammation, macrophage infiltration, tissue factor expression, and angiogenesis, several features of plaque vulnerability are fulfilled. It is therefore reasonable to conclude that JCAD predisposes individuals to a higher prevalence of vulnerable plaques and increased MI risk.

Targeting *JCAD* may thus represent a promising novel strategy for primary and secondary prevention of MI in the future.

## 6. Limitations and Future Directions

This study has several limitations. Its retrospective, cross-sectional design precludes establishing causal inference, and prospective studies are needed to confirm the predictive value of *JCAD* rs3739998. First, by design we studied MI risk within T2DM; an MI-without-T2DM comparator cohort was not available. Consequently, our data cannot disentangle whether rs3739998 primarily marks MI risk in general or a diabetes-specific susceptibility background; replication in independent MI-only cohorts is warranted. Furthermore, the analysis was restricted to a relatively homogeneous Slovenian cohort, which may limit generalizability to other populations. Replication in larger and ethnically diverse cohorts will be essential. We also focused solely on a single-nucleotide polymorphism, rs3739998, although additional *JCAD* variants may contribute to cardiovascular risk, and fine-mapping of the locus could provide further insights. Precision in multivariable estimates may be limited by correlations among included clinical covariates, which can widen confidence intervals even when point estimates increase after deconfounding. Additionally, since control status was determined using clinical criteria without requiring imaging, subclinical atherosclerosis in controls cannot be ruled out. Finally, while our findings suggest a biological role for *JCAD*, functional validation of how this polymorphism alters gene expression or endothelial activity was beyond the scope of this study.

Future research should therefore pursue replication studies across multiple populations, supported by functional assays in cellular and animal models to clarify the mechanistic pathways by which rs3739998 influences endothelial signaling, vascular remodeling, and thrombosis. Integrating *JCAD* into polygenic risk scores tailored to diabetic populations could refine cardiovascular risk prediction, while prospective trials are needed to test whether genetic screening for *JCAD* enhances clinical outcomes when combined with conventional risk assessment.

## 7. Conclusions

In a nationwide cohort of 1471 Slovenian patients with T2DM, we observed that the G allele and GG genotype of *JCAD* rs3739998 were more common among individuals with prior MI, yielding OR 1.18 and OR 1.37 (both *p* = 0.05). The direction and magnitude of effect align with prior genetic evidence implicating *JCAD* in coronary risk. In a CCTA substudy, rs3739998 did not associate with vessel count, stenosis severity, or CAC, suggesting that predisposition to MI conferred by *JCAD* may relate more to plaque vulnerability or thrombosis-prone environment than to overall calcified plaque burden.

These findings support *JCAD* rs3739998 as a potential biomarker for MI susceptibility in T2DM and encourage further validation in larger, diverse cohorts. Functional research is needed to understand how rs3739998 affects endothelial signaling and plaque vulnerability. Including *JCAD* in polygenic risk models tailored for diabetes could improve risk assessment and inform preventive measures.

## Figures and Tables

**Table 1:** Demographic and clinical characteristics of cases (MI patients) and controls in the Slovenian T2DM population.

Parameter	MI Patients (*n* = 387)	Controls (*n* = 1084)	*p*-Value
Age (years)	65.20 ± 9.87	64.35 ± 9.15	0.159
Male sex	237 (61.2%)	544 (50.2%)	**<0.001**
BMI (kg/m^2^)	29.67 ± 4.14	29.89 ± 4.84	0.428
Waist circumference (cm)	105.55 ± 11.17	107.96 ± 12.53	**0.016**
Systolic BP (mmHg)	147.31 ± 19.43	148.38 ± 20.91	0.404
Diastolic BP (mmHg)	81.56 ± 10.31	84.38 ± 10.47	**<0.001**
Fasting glucose (mmol/L)	8.99 ± 3.03	8.57 ± 2.55	**0.026**
Diabetes duration (years)	12.8 [10.0–23.0]	15.3 [10.0–20.0]	**<0.001**
HbA1c (%)	7.95 ± 1.32	7.77 ± 1.35	0.076
Total cholesterol (mmol/L)	5.05 ± 1.47	4.86 ± 1.20	**0.018**
HDL (mmol/L)	1.13 ± 0.30	1.23 ± 0.35	**<0.001**
LDL (mmol/L)	2.98 ± 1.25	2.79 ± 0.96	**0.003**
Triglycerides (mmol/L)	2.25 ± 1.73	2.08 ± 1.45	0.080
hsCRP (mg/L)	2.08 ± 1.29	2.08 ± 1.23	0.970
Current smokers	52 (13.4%)	108 (10.0%)	0.060
Arterial hypertension	326 (84.2%)	873 (80.5%)	0.107
Physical activity (>3×/week)	306 (79.1%)	729 (67.3%)	**<0.001**
Stroke	49 (12.7%)	80 (7.4%)	**0.002**
TIA	20 (5.2%)	36 (3.3%)	0.103
Therapy: Statins	306 (79.1%)	734 (67.7%)	**<0.001**
Therapy: Lipid-lowering drugs	312 (80.6%)	741 (68.4%)	**<0.001**
Therapy: Oral antidiabetics	131 (34.9%)	503 (46.4%)	**<0.001**
Therapy: Insulin	180 (46.5%)	626 (57.7%)	**<0.001**

Bold *p*-values denote statistical significance. BMI—body mass index; HbA1c—glycated hemoglobin; HDL—high-density lipoprotein; hsCRP—high-sensitivity C-reactive protein; LDL—low-density lipoprotein; MI—myocardial infarction; T2DM—type 2 diabetes mellitus; TIA—transient ischemic attack.

**Table 2:** Demographic and clinical characteristics of patients who underwent CCTA.

Parameter	Patients with CCTA (*n* = 146)
Age (years)	67.79 ± 8.97
Male sex	102 (69.9%)
BMI (kg/m^2^)	29.46 ± 3.89
Waist circumference (cm)	106.28 ± 10.78
Systolic BP (mmHg)	146.78 ± 19.64
Diastolic BP (mmHg)	80.67 ± 9.94
Fasting glucose (mmol/L)	8.80 [6.88–10.20]
Diabetes duration (years)	13.3 [10.0–16.0]
HbA1c (%)	7.85 ± 1.17
Total cholesterol (mmol/L)	4.65 [3.90–5.57]
HDL (mmol/L)	1.10 [1.00–1.30]
LDL (mmol/L)	2.60 [2.10–3.45]
Triglycerides (mmol/L)	1.80 [1.20–2.50]
hsCRP (mg/L)	1.90 [1.30–3.00]
Current smokers	33 (22.6%)
Arterial hypertension	129 (88.4%)
Physical activity (>3×/week)	115 (78.8%)
Stroke	13 (8.9%)
TIA	5 (3.4%)
Therapy: Statins	111 (76.0%)
Therapy: Lipid-lowering drugs	116 (79.5%)
Therapy: Oral antidiabetics	59 (40.4%)
Therapy: Insulin	82 (56.2%)

BMI—body mass index; HbA1c—glycated hemoglobin; HDL—high-density lipoprotein; hsCRP—high-sensitivity C-reactive protein; LDL—low-density lipoprotein; TIA—transient ischemic attack.

**Table 3:** Distribution of genotypes and allele frequencies of the JCAD rs3739998 polymorphism.

rs3739998	MI Patients (*n* = 387)	Controls (*n* = 1084)	*p*-Value	OR	*p*-Value (OR)
Genotype					
GG	102 (26.4%)	235 (21.7%)	0.14	1.37	**0.05**
CG	179 (46.3%)	514 (47.4%)		1.10	0.50
CC *	106 (27.4%)	335 (30.9%)		*	
Allele					
G (%) (MAF)	383 (49.5%)	984 (45.4%)	0.05	1.18	**0.05**
C (%)	391 (50.5%)	1184 (54.6%)		*	
HWE *p*-value	0.141	0.152			

* Reference; HWE—Hardy–Weinberg equilibrium; MAF—minor allele frequency; MI—myocardial infarction; OR—odds ratio. Bold *p*-values denote statistical significance.

**Table 4:** Binary logistic regression analysis of *JCAD* rs3739998 and MI occurrence in Slovenian T2DM patients.

Genetic Model	rs3739998 (MI/Controls)	aOR (95% CI)	*p*-Value
Codominant			
GG vs. CC *	102/235 vs. 106/335	1.63 (0.93–2.87)	0.09
CG vs. CC *	179/514 vs. 106/335	1.22 (0.76–1.99)	0.41
Dominant			
[GG + CG] vs. CC *	281/749 vs. 106/335	1.35 (0.87–2.11)	0.18
Recessive			
GG vs. [CG + CC]	102/235 vs. 285/849	1.34 (0.83–2.16)	0.22

* Reference; aOR—adjusted odds ratio; MI—myocardial infarction; T2DM—type 2 diabetes mellitus.

**Table 5:** Distribution of *JCAD* rs3739998 genotypes and alleles in 146 T2DM patients according to the number of diseased coronary arteries.

rs3739998	0 Arteries (*n* = 22)	1 Artery (*n* = 46)	2 Arteries (*n* = 30)	3 Arteries (*n* = 48)	*p*-Value
Genotype					
GG (MAF)	5 (22.7%)	11 (23.9%)	7 (23.3%)	10 (20.8%)	0.426
CG	12 (54.5%)	19 (41.3%)	10 (33.3%)	27 (56.2%)	
CC	5 (22.7%)	16 (34.8%)	13 (43.3%)	11 (22.9%)	
Allele					
G	22 (50.0%)	41 (44.6%)	24 (40.0%)	47 (49.0%)	0.667
C	22 (50.0%)	51 (55.4%)	36 (60.0%)	49 (51.0%)	

T2DM—type 2 diabetes mellitus; MAF—minor allele frequency. CCTA analyses interrogated anatomical burden across rs3739998 genotypes within T2DM; they were not stratified by prevalent MI status.

**Table 6:** Distribution of *JCAD* rs3739998 genotypes and alleles in 146 T2DM patients (292 alleles) according to the percentage reduction in coronary artery lumen.

rs3739998	<50% (*n* = 22)	≥50–≤75% (*n* = 70)	>75% (*n* = 54)	*p*-Value
Genotype				
GG	5 (22.7%)	16 (22.9%)	12 (22.2%)	0.546
CG	12 (54.5%)	28 (40.0%)	28 (51.9%)	
CC	5 (22.7%)	26 (37.1%)	14 (25.9%)	
Allele				
G	22 (50.0%)	60 (42.9%)	52 (48.1%)	0.595
C	22 (50.0%)	80 (57.1%)	56 (51.9%)	

Individuals (*n* = 146); total alleles = 292. T2DM—type 2 diabetes mellitus. CCTA analyses interrogated anatomical burden across rs3739998 genotypes within T2DM; they were not stratified by prevalent MI status.

**Table 7:** CAC scores according to genotype or allele of JCAD rs3739998 in 146 T2DM patients (292 alleles).

rs3739998	Median CAC (IQR)	*p*-Value
Genotype		
GG (*n* = 33)	342.4 (280.3–394.4)	0.42
CG (*n* = 68)	326.6 (249.6–388.2)	
CC (*n* = 45)	307.2 (259.8–347.4)	
Allele		
G (*n* = 134)	313.5 (232.1–370.0)	0.35
C (*n* = 158)	291.0 (232.7–347.9)	

Individuals (*n* = 146); total alleles = 292. CAC—coronary artery calcification; T2DM—type 2 diabetes mellitus.

## Data Availability

Data supporting reported results can be found locally in the Institute of Histology and Embryology, Faculty of Medicine, University of Ljubljana, Korytkova 2, 1105 Ljubljana, Slovenia.

## Abbreviations

The following abbreviations are used in this manuscript:

CAC	Coronary artery calcium
CCTA	Coronary computed tomographic angiography
T2DM	Type 2 diabetes mellitus
LDL	Low-density lipoprotein
GWAS	Genome-wide association studies
JCAD	Junctional Cadherin 5 Associated
MI	Myocardial infarction
BMI	Body mass index
HbA1c	Glycated hemoglobin
hsCRP	High-sensitivity C-reactive protein
CAD	Coronary artery disease
LMCA	Left main coronary artery
LAD	Left anterior descending artery
LCx	Left circumflex artery
RCA	Right coronary artery
HU	Hounsfield units
HDL	High-density lipoprotein
HWE	Hardy–Weinberg equilibrium
OR	Odds ratio
aOR	Adjusted odds ratio
